# A Busy Doctor’s Surgical Medley

**Published:** 1916-10

**Authors:** Neal Watt

**Affiliations:** Austin, Texas


### A Busy Doctor's Surgical Medley.

BY DR. NEAL WATT, AUSTIN, TEXAS.

Diversification of thought is healthful as well as appropriate to the calling of the general practitioner of medicine. His pursuit is an unending evolution of variegated experiences—a gastroenteritis, a typhoid case, a subject of pneumonia, malarial intricacies, rheumatic labyrinths, alternating and concurring with post-partum hemorrhage, placenta previa, transverse presentation, appendectomy, tumor laparotomies, intestinal surgery, etc., in a kind of kaleidoscopic showing.

I shall then present my thoughts on this occasion without any effort at classification. I will serve a hash, rather than the proverbial steak.

Case 1.—Female, col., ectopic pregnancy. Family history: negative. Past history, usual diseases of childhood. Patient denied having had either type of venereal disease. Menstrual history: began menstruating at thirteen, twenty-eight-day type, three to four days duration; no dysmenorrhea; married at age of nineteen; two children; normal labors; no miscarriages. Present trouble began about 4 o'clock in morning with an excruciating pain in right inguinal region, radiating down right thigh. Patient in state of shock bordering on collapse. Rapid, thready pulse; complained of chilliness. She had a cold, clammy perspiration, excessive vomiting and a temperature of 100. I evidently had here symptoms of an abdominal calamity. I advised immediate removal to the hospital. Here further examination showed a focusing of tenderness and pain over McBurney and Morris' points. Vaginal examination disclosed a small mass on right side and a little bloody ooze. Patient denied the possibility of pregnancy. Blood count—total 14,600 whites with 90 per cent polymorphonuclears. I diagnosed acute appendicitis and advised an immediate operation, which was promptly and positively declined. She returned home, and ten days later at night her husband called me up and asked me to come out at once, saying that his wife was suffering intensely. I asked him if he was ready to have her operated on, and he said he did not know. I then refused to go. Forty-eight hours later he rang me up and said that he was bringing his wife to the hospital for an operation. I next saw her in the operating room. Pulse was fast and small; abdomen distended, and pain and tenderness diffused over the entire area. Patient was in an extremely bad state. On opening the abdomen we found it filled with blood and the pelvis with blood clots. Further examination showed an ectopic pregnancy of right tube with rupture. The bleeding was arrested, and as the left tube and ovary were diseased a supra vaginal hysterectomy was done. Patient made a slow but good recovery.

Case 2.—Pyloric stenosis, due to chronic ulcer. White, male, age about fifty-five. Family history good. Parents lived to good ripe age, and brothers and sisters advanced in years, are all in good health. Personal history: No venereal disease; habits temperate; has used both alcoholic drinks and tobacco, but in moderation. Was well and strong until about four years ago. His trouble started with stomach disturbances. The usual symptoms of so-called indigestion and pain almost from the start, but no early vomiting. The gastrodynia was progressive in intensity and indiffusibility. It radiated mostly to right, at times focusing in close vicinity to the gall bladder. Again it centered to the left of cardiac end of stomach. At this time there was some jaundice present with localized tenderness over region of gall bladder. Gall stones were diagnosed and he was operated for same by a local surgeon but none were found. The gall bladder was drained and the patient made an uneventful recovery from the operation. However, this did not relieve his condition. The pains grew worse. He suffered continuously around the region of his stomach. He complained of being hungry, always wanting food—would eat heartily and then in about three hours the pain would get severe and vomiting would take place, expelling the whole contents in a partially digested condition. This would give him a little relief for the time being and then the pains would set up again, which were not wholly but partially relieved by eating. His food assimilating power dwindled down to nil; he grew rapidly weaker, showing marked emaciation, and in fact was starving to death. That a pyloric blocking by growth or stricture existed seemed clear, but the exact causative factor was enshrouded in obscurity and uncertainty. An operation was advised and assented to. On opening the abdomen we found the omentum adhered to the abdominal wall all around the gall bladder; the gall bladder itself contracted and non-inflamed. There was a mass of dense adhesions around the pyloric end of stomach and upper duodenum—the stomach being adhered to liver. The pyloric opening was contracted and almost closed, showing presence of an ulcer and old scar tissue. After breaking up as many adhesions as we deemed advisable, we did a posterior gastro-jejunostomy, ignoring the affected end of the stomach. The post-operative treatment was simple. There was very little vomiting, and this was mostly from the ether, occurring in the first twenty-four hours. We began feeding him liquids on the third day, and at the end of the week he was taking soft diet, which was readily digested. The patient was able to leave the hospital at the end of second week, and at end of third week was able to be out. Now, two months later, the pains have entirely disappeared and the patient is eating three hearty meals a day with absolutely no gastric disturbances. He is rapidly regaining weight and strength.

CASE 3.—Gunshot wound of right kidney and liver. Patient, F. K., age 11, white, male. Accidentally shot by playmate with .22-caliber target rifle; the bullet entering two inches to right of spinal column, opposite the second lumbar vertebra, ranging outward and upward. Patient was also shot through metacarpo phalangeal joint of thumb of right hand, he having instinctively thrown his hand behind him, as he turned, when his playmate pushed the cocked gun at him, the end of the weapon striking his hand first as it exploded. We first saw him about three hours after the accident occurred. He then complained of pain in right lumbar region. There was some distension and rigidity of abdomen, pulse fast and rather thready. From location of bullet wound and outward signs we suspected kidney and intestinal perforation. Catheterization showed the urine filled with blood. Operation was immediately resorted to. Right rectus incision was made, abdomen found filled with blood and blood clots. This was rapidly sponged out and careful examination revealed no intestinal perforation. There was free hemorrhage from the region of right kidney. Further examination showed bullet wound of liver with free bleeding. This was packed with gauze and as the kindey was easily reached from front, the posterior peritoneum was opened and the kidney perforation packed with idoform gauze. A stab wound was made posteriorly and the end of the gauze and the cigarette drain were brought out through this. The posterior peritoneum was then sutured back over the kidney, the latter not being moved from its bed. The gauze packing in and around the liver opening was brought out of the abdominal wound, a drainage tube also being inserted in pelvis.

Post-operative treatment: Patient was kept in the Fowler position for seventy-two hours. In place of saline proctoclysis we used sterile water 12 ounces, whisky 2 ounces, every six hours. Gauze and tubes removed on the third day except the pelvic drain, which was removed the fourth day. The wound of hand, more particularly on account of the powder burn at site of traumatism, was treated with anti-tetanic serum 1500 units, as well as the ordinary surgical measures. This was well, for about the seventh day the boy's fever suddenly went up to 102$\frac{2}{5}$. There was a severe axillary adenitis with other untoward symptoms, including a red streak up forearm, soreness in flexor muscle tendons. Pus was found in hand, evacuated and drainage of wound established. Patient at end of twelve days is apparently well on the road to recovery.

